# A Review on Non-Enzymatic Electrochemical Biosensors of Glucose Using Carbon Nanofiber Nanocomposites

**DOI:** 10.3390/bios12111004

**Published:** 2022-11-11

**Authors:** Ali Mohammadpour-Haratbar, Saeid Mohammadpour-Haratbar, Yasser Zare, Kyong Yop Rhee, Soo-Jin Park

**Affiliations:** 1 Biomaterials and Tissue Engineering Research Group, Department of Interdisciplinary Technologies, Breast Cancer Research Center, Motamed Cancer Institute, ACECR, Tehran 1949635881, Iran; 2 Faculty of Sciences, Urmia University, Urmia 5756151818, Iran; 3 Department of Mechanical Engineering (BK21 Four), College of Engineering, Kyung Hee University, Yongin 17104, Korea; 4 Department of Chemistry, Inha University, Incheon 22212, Korea

**Keywords:** carbon nanofiber, electrochemical biosensors, electrodes, nanocomposite, nanoparticles

## Abstract

Diabetes mellitus has become a worldwide epidemic, and it is expected to become the seventh leading cause of death by 2030. In response to the increasing number of diabetes patients worldwide, glucose biosensors with high sensitivity and selectivity have been developed for rapid detection. The selectivity, high sensitivity, simplicity, and quick response of electrochemical biosensors have made them a popular choice in recent years. This review summarizes the recent developments in electrodes for non-enzymatic glucose detection using carbon nanofiber (CNF)-based nanocomposites. The electrochemical performance and limitations of enzymatic and non-enzymatic glucose biosensors are reviewed. Then, the recent developments in non-enzymatic glucose biosensors using CNF composites are discussed. The final section of the review provides a summary of the challenges and perspectives, for progress in non-enzymatic glucose biosensors.

## 1. Introduction

In recent decades, diabetes mellitus has become a worldwide epidemic [1]. Globally, diabetes is expected to become the seventh leading cause of death by 2030, according to reports [2,3,4,5]. Generally, a blood glucose range of less than 100 mg/dL is considered normal. If the blood glucose level of a patient is higher than 126 mg/dL on two consecutive fasting blood glucose tests, the patient is considered diabetic [6,7]. In the human body, glucose levels can be categorized into two types. First, low glucose levels, known as hypoglycemia, can lead to fainting, comas, and even death, when blood sugar levels drop below normal levels. Hyperglycemia is the second phenomenon that undesirably influences the kidneys, eyes, nerves, and blood vessels, causing blood sugar levels to rise abnormally [8,9,10]. As a result of insufficient insulin production in the body (Type-1 diabetes) or not being able to use the insulin produced in the body (Type-2 diabetes), diabetes is defined by hyperglycemia, or a high blood glucose level [11,12,13]. The hormone insulin aids the cells of the body in absorbing glucose in the blood. Glucose concentrations in the blood can rise to a level where the kidneys, eyes, nerves, and heart can be damaged, in both Type-1 and Type-2 diabetes, if the body is not provided with insulin [14,15].

In response to the increasing number of diabetes patients worldwide, glucose biosensors with high sensitivity and selectivity have been developed for rapid detection [16,17]. Biosensors are devices that transform biological events into electrical signals [18,19,20]. A variety of techniques have been proposed for glucose monitoring, but electrochemical biosensors are preferred, owing to their ease of use, low cost, and ability to be combined with portable devices to provide numerical analysis [21,22,23,24]. Two kinds of electrochemical glucose biosensors are available: enzymatic and non-enzymatic. In an enzymatic biosensor, glucose oxidase enzyme (GOx) is used for the detection of glucose; whereas in non-enzymatic biosensors, the glucose detection mechanism is based on electrocatalytic activities [25,26,27,28].

Carbon nanofiber (CNF) is similar to CNTs in structure and properties, but it is easier to produce, has a lower cost, and provides improved functionality [29,30,31]. CNFs are carbon nanomaterials with diameter in the nanometer range and length in the micron range [32]. Much attention has been given to nanocomposites, owing to their exceptional properties [33,34,35,36]. Modification of bare electrode surfaces using nanocomposite materials comprising nanomaterials and matrices takes advantage of the properties of both nanomaterials and matrices [37,38,39]. Conductive nanocomposites are used in numerous biomedical applications, including biosensors and actuators [40,41,42]. CNFs have good thermal conductivity, outstanding electrical conductivity, high porosity, and high surface area, making them excellent substrates and supporting matrices for embedding nanoparticles for the synthesis of CNF-based nanocomposites [43,44,45]. CNFs are considered a strong substrate for non-enzymatic biosensors, because of their easy manufacturing process, high electrical conductivity, and high specific surface area. Additionally, CNF-based electrodes can be prepared as free-standing, without the addition of a binder or glassy carbon electrode (GCE) being applied to the actual glucometer as a strip [46,47].

**Figure 1:**
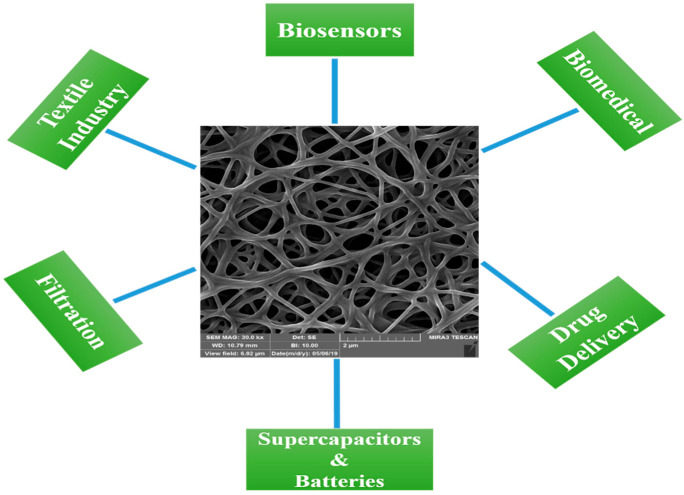
Different applications of CNFs.

Electrospinning and catalytic vapor deposition growth (CVD) are the two main methods for fabricating CNFs [48]. Electrospinning produces nanofibers by uniaxial stretching of a viscoelastic solution. Electrospinning uses electrostatic forces to stretch the solution as it solidifies, unlike dry-spinning and melt-spinning. Fibers are formed by drawing the solution, as long as the electrospinning jet is fed with a sufficient solution. Therefore, fiber formation is continuous, without interruption of the electrospinning jet [49]. Electrospinning has been used to produce nanofibers of various polymers, with diameters ranging from tens of nanometers to a few micrometers, and in various forms, such as nonwoven mats (webs) and yarns. A continuous nanofiber can be produced from polymer solutions or melts using this relatively simple and low-cost method [50]. Through the electrospinning technique, CNFs can be easily produced via the carbonization of polymer nanofibers, which are called electrospun CNFs (ECNFs) [32,51]. Polyacrylonitrile (PAN) is the most widely used polymer precursor for the electrospinning of carbon nanofibers, owing to its easy processing, excellent mechanical properties, and high carbon yield [52,53]. CNFs can also be made by direct carbonization of other polymers, such as cellulose, chitin, lignin, and chitosan NFs [54,55]. CVD can also produce carbon nanofibers with different structures and properties from those made by electrospinning, called vapor-grown carbon fibers (VGCF) [50]. In CVD, CNFs are synthesized using a catalyst to grow carbon in a 1D pattern on a substrate [56]. As a result of their high electrical conductivity, mechanical properties, and specific surface area, CNFs have numerous potential applications in a broad range of industries, such as energy storage devices (supercapacitors and batteries), the reinforcement of nanocomposites, biomedicine, textiles, filtration, drug delivery, and especially biosensing [57,58,59,60]. Figure 1 illustrates some applications of CNFs in different fields.

**Figure 2:**
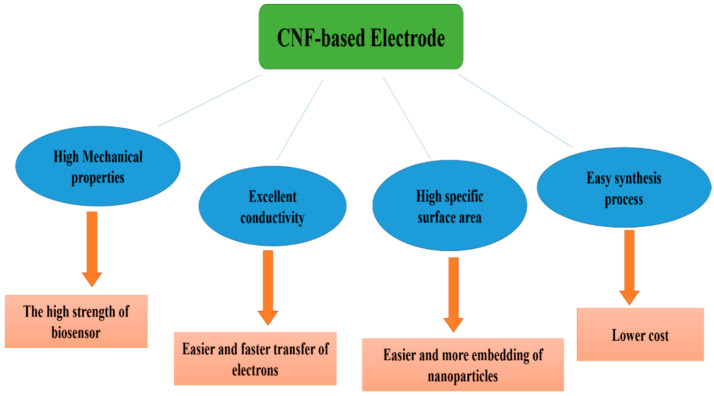
The characteristic properties of CNFs and the effect of the properties on the performance of the fabricated biosensor.

Figure 2 illustrates the characteristics of CNFs that make them ideal diagnostic electrodes for biosensors. Owing to their high conductivity, CNFs facilitate and accelerate electron transfer in the electrode, which results in higher biosensor sensitivity [60]. Additionally, CNF-based electrodes possess high mechanical properties, resulting in the high strength of the biosensor electrodes. Furthermore, carbon nanofibers are easy to synthesize and produce, which reduces the cost of manufacturing biosensor electrodes [61]. Moreover, owing to the large specific surface area of carbon nanofibers, a large number of nanoparticles can be embedded on their surface, with a uniform dispersion, thus enhancing the electrocatalytic properties of the carbon nanofiber electrodes and increasing the sensitivity of the biosensor [62].

After a brief discussion on enzymatic and non-enzymatic biosensors, non-enzymatic biosensors of glucose based on nanocomposites of CNF are discussed. Subsequently, the challenges associated with non-enzymatic glucose biosensors and their advantages are summarized.

## 2. Enzymatic Electrochemical Biosensors for Glucose Sensing

Glucose oxidase (GOx) is a common enzyme used in enzymatic biosensors [63,64,65]. In the 1960s, Clark and Lyons described the first enzyme-based biosensor [66]. Enzyme-based biosensors are widely used and extensively studied, owing to their excellent selectivity and sensitivity [67]. There are three types of enzymatic glucose biosensors [68,69]. In the first type, an amperometric technique is used, which involves immobilizing a catalytic enzyme, to reduce oxygen to hydrogen peroxide. Oxygen dependence and a high operating potential are the main disadvantages of this kind of biosensor [70,71]. The next type of glucose biosensor is designed to overcome the oxygen deficiency under a low oxygen pressure and high applied potential. GOx is immobilized on redox mediators in these biosensors [72,73]. Typical electron redox mediators include ferrocene derivatives, ferrocyanide, conducting organic salts, and quinones [74,75]. In the third type of biosensor, electrons are transferred directly between the immobilized enzyme active sites and the electrode surfaces [76]. Compared with the first and second types of glucose biosensors, the third type exhibits deteriorated detection properties, especially a smaller linear range [26,67]. The properties of GOx influence the performance of enzymatic biosensors. Studies have shown that GOx is unstable at pH values lower than 2 and higher than 8. Furthermore, humidity and temperatures above 40 °C significantly reduce the activity of enzymes. Moreover, enzyme immobilization methods are complex and costly [77,78]. Recent research has increasingly focused on non-enzymatic glucose biosensors, because of the disadvantages of GOx [79]. Non-enzymatic biosensors work by directly oxidizing glucose on an electrode that possesses electrocatalytic activity and contains a transition metal center [80,81].

## 3. Non-Enzymatic Electrochemical Biosensors for Glucose Sensing

Owing to the inherent disadvantages of traditional glucose enzymatic biosensors, such as their high fabrication costs, poor enzyme stability, pH value dependence, and other dedicated limitations, non-enzymatic glucose sensors are gaining increased attention. Considering the drawbacks of enzyme-based biosensors, researchers are developing new electrochemical biosensors without using any biological molecules such as enzymes; that is, non-enzymatic biosensors [82,83,84]. Unlike enzymatic sensors, non-enzymatic glucose biosensors do not suffer from enzyme immobilization problems. Furthermore, the stability of non-enzymatic biosensors is higher, owing to the absence of enzymes. An enhanced sensitivity and specificity of detection can be achieved through functionalized nanomaterials acting as catalysts or immobilization platforms. The use of enzyme-less materials based on straight glucose electro-oxidation led to the fourth-generation glucose biosensor [85,86]. Compared with their enzyme counterparts, these biosensors exhibit considerably enhanced electrocatalytic performance for glucose detection, owing to the incorporation of nanostructured metal or metal oxide on the electrode surfaces [87,88,89]. The key step in designing non-enzymatic biosensors is selecting the appropriate catalyst for glucose detection [90,91]. The sensitivity, selectivity, and stability of certain non-enzymatic electrochemical glucose biosensors have been well-established, through extensive research. In addition, non-enzymatic glucose biosensors are cost-effective, stable, reproducible, and simple to develop, without any complicated enzyme immobilization methods [92,93,94]. There are several types of non-enzymatic electro-catalysts, including metallic nanoparticles, metal oxide nanoparticles, bimetallic/alloys nanostructure, carbon nanomaterials, and metal/carbon nanomaterial-based nanocomposites [61,62,95,96].

## 4. Non-Enzymatic Glucose Biosensors Based on Nanocomposites of CNF

Recently, CNFs have been considered excellent substrates for embedding metal and metal oxide nanoparticles as sensitive nanocomposites for biosensors, owing to their high specific surface area and porosity, high mechanical properties, and high electrical conductivity [31,97,98,99]. Owing to advantages such as a facile and environmentally friendly fabrication process, high conductivity, high surface area, and high porosity, CNFs have been widely used as electrodes for biosensors. The large specific surface area of the CNF allows a large number of nanoparticles and catalysts to be incorporated onto its surface, enhancing the electrocatalytic performance when reacting with a target analyte [100,101]. Moreover, adding metal nanoparticles or metal oxide nanoparticles to the CNF matrix increases the electrocatalytic activity of the biosensor and enhances the biosensor performance, in terms of the limit of detection (LOD), linear range (LR), and sensitivity.

With the growing number of diabetic patients, it is necessary to develop a blood glucose measuring device. Commercial glucometer strips are usually coated with enzymes. The instability of enzymes in environmental conditions, the difficulty of immobilizing enzymes on electrode surfaces, and the high cost of enzymes have motivated researchers to develop non-enzymatic biosensors. A substrate is used to embed the catalyst in non-enzymatic biosensors. Carbon nanofibers (CNFs) are a good substrate for non-enzymatic biosensors, because of their easy manufacturing process, high electrical conductivity, and high specific surface area. Additionally, CNF-based electrodes can be prepared free-standing, without the addition of a binder or GCE, to use with the actual glucometer as a strip. Various nanostructures have been attached to the CNF surface as electro-catalysts, including noble metals, nickel-based nanoparticles, cobalt-based nanoparticles, and copper-based nanoparticles [102,103,104,105]. Table 1 summarizes all the non-enzymatic glucose biosensors that use CNF composite electrodes.

### 4.1. Mono Metallic (Metal Oxide)/CNF Nanocomposites as Non-Enzymatic Glucose Biosensors

Non-enzymatic biosensors with CNF nanocomposites are typically made using nickel, cobalt, copper, platinum, nickel oxide, cobalt oxide, and copper oxide nanoparticles. Liu et al. [122] proposed a glucose biosensor with Ni nanoparticle-loaded CNF electrodes by combining electrospinning with thermal treatment. Owing to the large surface of CNF and the high electrocatalytic activity of Ni nanoparticles, the Ni/CNF electrode showed an excellent electrocatalytic performance for oxidizing glucose.

**Figure 3:**
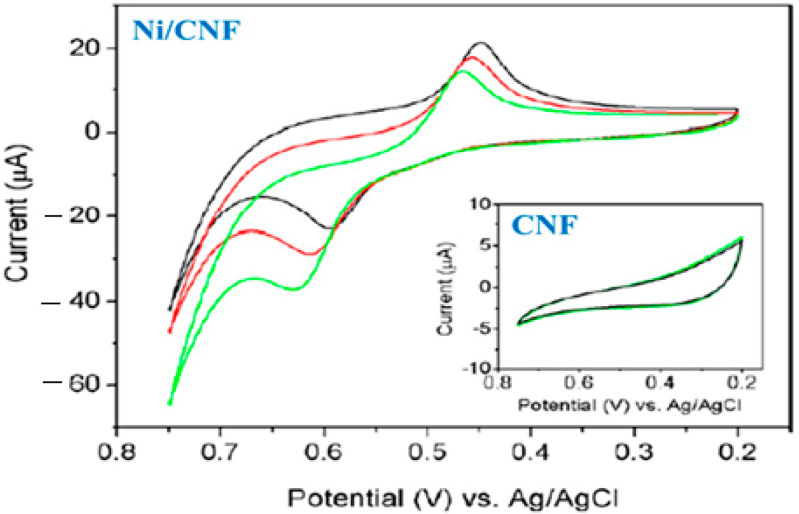
CVs of the Ni/CNF nanocomposite without (dark line) and with 2 mM (red line) and 4 mM (green line) glucose. Inset shows the CVs of pure CNF without (dark line) and with glucose. Reprinted from Ref. [122], with permission from Elsevier.

As shown in Figure 3, the Ni/CNF electrode exhibits a pair of redox peaks in the blank NaOH solution, corresponding to the Ni(II)/Ni(III) redox couple. An increase in the glucose concentration also led to a greater voltammetric response. As a result, Ni/CNF electrodes showed an adequate electrocatalytic activity. Additionally, the CNF electrode showed no response current to glucose, whereas the Ni/CNF electrode showed oxidation/reduction peaks, indicating that Ni nanoparticles play a key role in electrocatalytic sensing [133]. The figure also shows that the peak current in the Ni/CNF nanocomposite electrode changed with the glucose concentration, indicating that the biosensor is highly sensitive. Moreover, the results indicate that Ni/CNF electrodes do not exhibit significant changes in their current response when glucose is oxidized, which indicates that the electrode is highly resistant to surface fouling and has excellent selectivity, making it a good candidate for developing a low-cost non-enzymatic glucose biosensor. The presented sensor has a sensitivity of 420.4 μA·mM^−1^·cm^−2^, an LOD of 10^−3^ mM, and an LR of 2 × 10^−3^–2.5 mM.

**Figure 4:**
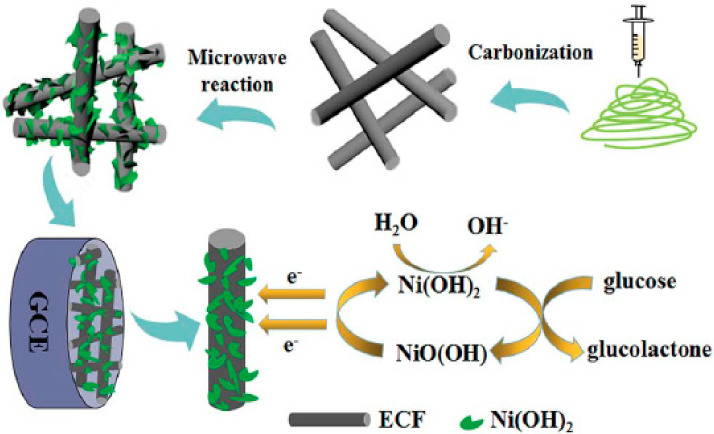
Ni(OH)_2_/ECNF nanocomposite fabrication steps. Reprinted from reference [106], with permission from the Royal Society of Chemistry.

Biosensors have been constructed using a variety of nanostructures, including nanoplatelets, nanoflakes, nanorods, nanoribbons, nanowires, and nanotubes because of their interesting properties, such as a large surface-to-volume ratio, high porosity, excellent electrocatalytic properties, and high surface area [24,134,135]. Chen et al. [106] constructed a biosensor using a nanocomposite of Ni(OH)_2_ nanoplatelets and ECNF. Figure 4 shows the steps involved in manufacturing Ni(OH)_2_/ECNF nanocomposites. Initially, CNFs are synthesized using an electrospinning technique, and then Ni(OH)_2_ nanoplatelets are deposited on the surface of CNFs. A conductive substrate such as CNF facilitates the electron transfer and improves the sensitivity of biosensors. CNFs are also capable of embedding more nanoparticles with good dispersion, owing to their large specific surface area. In addition, Ni(OH)_2_ with the morphology of nanoplatelets increases the effective specific surface area of the electrode and results in an easier absorption of glucose molecules on the electrode surface, thereby improving the performance of the biosensor. EIS analysis of this group showed that Ni(OH)_2_/ECNF-modified nanocomposites had smaller R_ct_ values than pure Ni(OH)_2_, suggesting that the ECNF substrate considerably enhanced the conductivity of the nanocomposite.

Ni(OH)_2_/ECNF electrodes displayed smaller anodic peak potentials and higher peak currents than pure Ni(OH)_2_ electrodes, suggesting that the high electrical conductivity of the ECNF matrix facilitated the redox processes of Ni(II)/Ni(III). Furthermore, one-dimensional ECNFs can prevent aggregation of Ni(OH)_2_ nanoparticles and may simplify the diffusion of the electrolyte into Ni(OH)_2_ nanoplatelet electrodes. Thus, when the excellent electrochemical performance of ECNFs, such as their high conductivity and high surface area, is combined with the electrocatalytic properties of Ni(OH)_2_ nanoplatelets, a synergistic effect is achieved.

**Figure 5:**
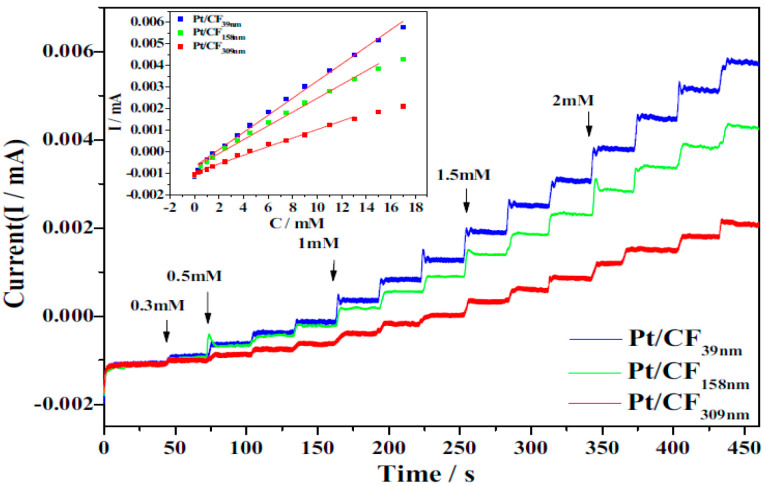
Amperometric analysis and corresponding calibration plots (inset) of Pt/ECNF nanocomposites with different diameters of ECNF. Reprinted from reference [115], with permission from Elsevier.

Nanofillers with small diameters (d) and long lengths (l) have a high aspect ratio (l/d) and large specific surface area; hence, they have excellent electron-transfer properties and electrocatalytic activity [136]. An enzyme-less glucose biosensor was developed by Ye et al. [115] using ECNFs with various diameters as Pt-catalyst supports. In ECNFs with a smaller diameter, more Pt atoms are observed on the surface of the ECNF, owing to the high surface area. In addition, a ECNF with a smaller diameter has a greater electrochemical activity and superior electrocatalytic performance for the detection of glucose. Thus, a Pt/ECNF nanocomposite electrode with a smaller ECNF diameter shows a better response current and higher sensitivity (Figure 5). For nanofibers with smaller diameters, the slope of the calibration curve is higher, indicating that the sensitivity increases with the decreasing diameter of the CNFs. In other words, reducing the fiber diameter increases the aspect ratio, which improves the properties, especially the electrical conductivity. Carbon nanofibers with higher electrical conductivity result in a faster electron transfer on the electrode surface, resulting in improved biosensor sensitivity and response times. In addition, according to the chronoamperometric diagram, each time glucose is added and a step is formed, the current remains constant, until the next glucose injection, showing the high stability of the biosensors.

**Figure 6:**
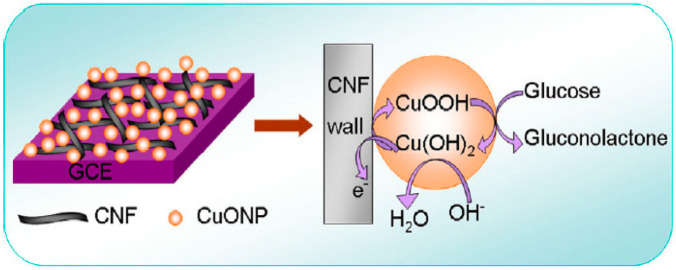
Schematic illustration of glucose recognition mechanism based on a CuONPs-CNF nanocomposite. Reprinted from reference [117], with permission from Elsevier.

Zhang et al. [117] presented a nanocomposite of nano-cupric oxide (CuONPs) on a CNF surface for fabricating a non-enzymatic glucose biosensor. Figure 6 illustrates the glucose detection mechanism of a CuONPs-CNF nanocomposite. Glucose was rapidly oxidized to gluconolactone by the copper (III) formed on the surface of the nanoparticles. Meanwhile, the oxidation peak current increased, whereas the reduction peak current decreased, owing to the feeding of Cu (III) agents and the formation of Cu (II) agents. Cu (III) may act as a mediator for electron transfer. CuONPs electrocatalysts and glucose molecules undergo an electrochemical reaction, releasing electrons and producing electrical signals proportional to the glucose concentration. The conductive substrate of CNFs promotes a faster electron transfer, enables CuONPs to disperse on their surface, and, as a supporting matrix, prevents the separation of nanoparticles from the electrode surfaces. The results of this study indicate that CuONPs can act as a strong electrocatalyst for glucose oxidation, thus enhancing the performance of the biosensor. The designed biosensor revealed a wide LR to glucose from 5 × 10^−4^ mM to 11.1 mM, with a high sensitivity of 2739 μA·mM^−1^·cm^−2^ and a low LOD of 2 × 10^−4^ mM.

### 4.2. Hybrid Nanoparticle/CNF Nanocomposites as a Non-Enzymatic Glucose Biosensor

Researchers have focused on creating hybrid nanostructures composed of two or more different materials, to overcome the limitations of single-component nanoparticles, improve their properties, achieve novel properties that are not possible with single-component nanoparticles, and achieve multiple functionalities. Nanostructures with hybrid compositions have superior electrochemical properties than nanoparticles with single components. There is a synergistic effect between the components, which produces these results. Consequently, it is expected that combining two or more nanoparticles with ECNF matrices could be a unique approach for fabricating advanced electrode materials for non-enzymatic glucose detection [60,137,138].

Ding et al. [107] developed a nanocomposite of CoFe_2_O_4_ nanoparticles and ECNFs via electrospinning and subsequent heat treatment process. ECNFs with outstanding conductivity functioned as a substrate guiding the development of CoFe_2_O_4_ particles, thus improving the CoFe_2_O_4_ dispersion and forming a superior electron transportation path. Additionally, electrospun nanofibers can be used in the electrocatalytic reaction processes of CoFe_2_O_4_ as electron transport channels, owing to their large surface, electrical conductivity, and broad electrochemical window. The proposed nanocomposite electrode exhibits excellent detection properties, owing to the effects of CoFe_2_O_4_ nanoparticles and an ECNF matrix. It has an adequate sensitivity of 318 μA·mM^−1^·cm^−2^, a wide LR from 10^−2^ to 3.52 mM, and a low LOD of 3.25 × 10^−4^ mM.

**Figure 7:**
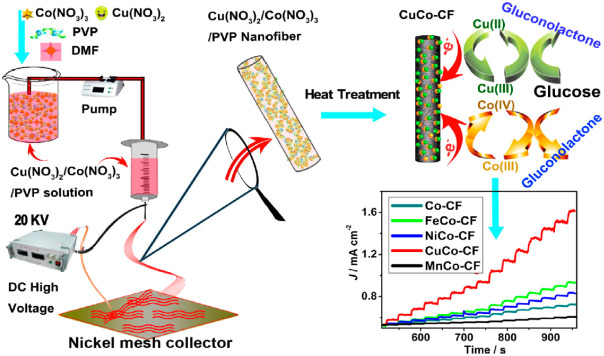
Synthesis steps of Co/CNFs and MCo/CNFs (M = Fe, Ni, Cu, and Mn) nanocomposites, electrochemical mechanism of electrodes, and the results of amperometric analysis of biosensors. Reprinted from reference [112], with permission from Elsevier.

Li et al. [112] prepared MCo (M = Cu, Fe, Ni, and Mn) nanoparticles embedded in CNFs as non-enzymatic glucose biosensors. Figure 7 represents the stages of the synthesis of nanocomposites and the fabrication of the biosensors. As shown in the figure, during the process of heat treatment, the PVP nanofibers are converted into CNFs, and at high temperatures, metal nanoparticles diffuse from the bulk of the CNFs to their surface, owing to the presence of a metal concentration gradient between the bulk and CNF surface [60,61]. The presence of nanoparticles on the surface of CNFs improves the electrocatalytic properties of the electrode surface in the reaction with glucose analyte and enhances the performance of the biosensor. Based on these results, CuCo/CNFs exhibit the highest catalytic ability, followed by Co/CNFs, NiCo/CNFs, FeCo/CNFs, and MnCo/CNFs. Additionally, according to the chronoamperometric plot shown in Figure 7, CuCo/CNFs display the highest sensing efficiency, even for the sensing of glucose in a real sample, owing to the structural advantages of the CNF matrix and the simultaneous effects of Co (III)/Co (IV) and Cu (II)/Cu (III) redox couples. The proposed biosensor has an LOD of 10^−3^ mM, high sensitivity of 507 μA·mM^−1^·cm^−2^, and an LR of 0.02–11 mM. Moreover, the proposed biosensor is neutral with other interfering species in blood and exhibits a high selectivity.

**Figure 8:**
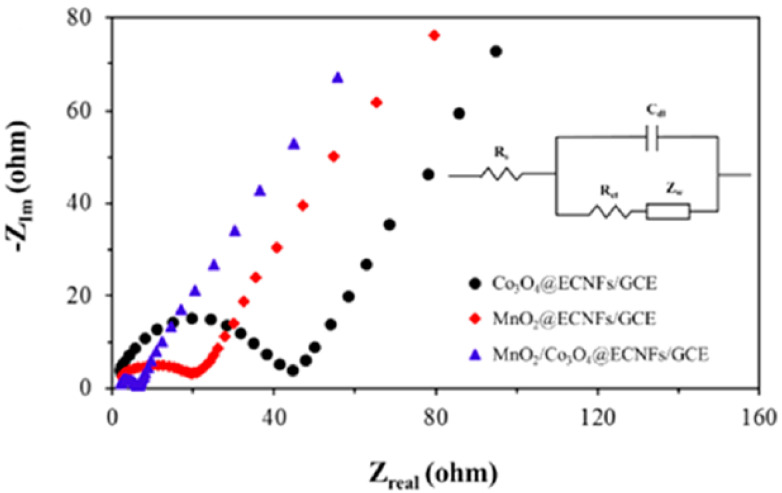
Nyquist plots of electrodes in a 0.1 M KCl solution containing 5 mM [Fe(CN)_6_]^3−/4−^. Reprinted from reference [116], with permission from ACS.

Yin et al. [116] developed an enzyme-less biosensor by decorating the surface of an ECNF matrix with binary nanoparticles of Co_3_O_4_ and MnO_2_ for glucose sensing. Compared with monometallic Co_3_O_4_ or MnO_2_ electrodes with ECNFs, binary MnO_2_-Co_3_O_4_/ECNF electrodes showed an outstanding consistency, with high porosity, large electrochemical surface area, increased conductivity, and high glucose electro-oxidation effectiveness. As shown in the EIS spectra in Figure 8, MnO_2_-Co_3_O_4_/ECNF electrodes exhibited the smallest Nyquist semicircle and the lowest R_ct_ compared with ECNF electrodes electrodeposited with MnO_2_ or Co_3_O_4_. This indicates that the MnO_2_/Co_3_O_4_@ECNF electrodes exhibited the highest electron and mass transport efficiency. The permeable structure with a bigger surface for electrolyte ions could explain this decrease in resistance. Owing to the morphological and physical aspects of binary MnO_2_-Co_3_O_4_/ECNFs, the ion penetration to the electrode surface and conductivity improved. The binary metal oxides, as well as the ECNF matrix with high porosity, considerably enhanced the detection performance, with an outstanding sensitivity = 1159 μA·mM^−1^·cm^−2^ and a low LOD = 3 × 10^−4^ mM with LR = 0.005–10.9 mM.

**Figure 9:**
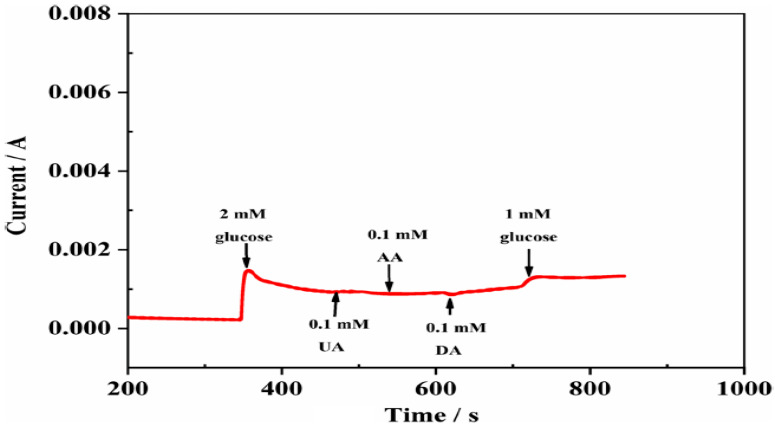
The amperometric response of an electrode to successive injections of 1.0 and 2.0 mM glucose and 0.1 mM interferents of UA, AA, and DA in 0.1 M NaOH solution. Reprinted from reference [61], with permission from Springer.

Recently, our group [61] presented a glucose biosensor using metal and metal oxide nanoparticles of nickel, cobalt, and hybrid nanoparticles decorated on the surface of ECNFs. As shown in the SEM images, the nanoparticles were uniformly dispersed on the ECNF surface. The SEM image also shows that carbon nanofibers with nanometer diameters and micron lengths were synthesized, without beads. The results showed that the nickel nanoparticles exhibited better electrocatalytic properties in reaction with glucose than cobalt nanoparticles. Nanocomposites containing nickel had a higher electrical conductivity than those containing cobalt. The increased electrical conductivity of nickel metal, compared with cobalt metal, increased the electron transfer in glucose oxidation-reduction reactions, leading to stronger electrocatalytic properties and better sensitivity in biosensors. The nickel electrode sensitivity was 610.6 μA·mM^−1^·cm^−2^, which was much higher than the cobalt electrode sensitivity (236.85 μA·mM^−1^·cm^−2^). The electrocatalytic properties of the final biosensor with the hybrid nanoparticles were improved by increasing the percentage of nickel in the electrospinning solution. Moreover, the ECNF matrix acted as an excellent supporting substrate for embedding nanoparticles because of its high conductivity and large surface area. As a result, the electrode displayed excellent selectivity for detecting glucose, even in the presence of interfering agents (Figure 9). This high selectivity may be attributed to the lower applied potentials, which could be achieved because of the higher electrical conductivity and easier electron transfer of CNF-based nanocomposites.

Huan et al. [127] developed a nanocomposite of CuSn/CNFs as a biosensor for glucose recognition. They found that an appropriate amount of Sn considerably increases the electrocatalytic activity of Cu/CNFs for oxidation of glucose. Thus, by adding doped Sn to CuSn/CNF, large defects can be introduced to increase the electron transference rate and decrease the activation energy needed for the intermediate process. Therefore, CuSn/CNFs exhibited a higher oxidation current than Cu/CNFs for the detection of glucose [139]. The interaction between Cu and Sn nanoparticles on the CNF matrix enhanced the electrocatalytic activity of CuSn/CNFs for sensing. The biosensor exhibited an exceptional sensing performance for glucose, with a broad LR in the range from 10^−4^ to 9 mM, low LOD of 8 × 10^−5^ mM, and adequate sensitivity of 291.4 μA·mM^−1^·cm^−2^.

## 5. Conclusions and Future Challenges

This review has presented an overview of the recent advances in non-enzymatic electrochemical biosensing of glucose using CNF-based nanocomposites. A wide range of electrochemical biosensors with excellent analytical performance have been constructed using the attractive properties of CNFs, such as their high porosity, excellent conductivity, great mechanical properties, and high superficial area. The high electrical conductivity of CNFs improves the performance of biosensors. Adding metal nanoparticles or metal oxide nanoparticles to the CNF matrix increases the electrocatalytic activity of the biosensor, as well as its performance. In the initial research studies, metals such as Ni, Cu, Co, Pt, and Pd, and compounds such as nickel oxides, cupric oxide, and cobalt oxides were embedded into a CNF matrix for manufacturing nanocomposite electrodes. More recently, combinations of different metal compounds, such as alloys and bimetallic nanoparticles embedded into CNFs, have been explored for their synergistic effects and excellent electrocatalytic performance. However, a major obstacle to using non-enzymatic biosensors for blood samples is that most non-enzymatic glucose biosensors cannot catalyze glucose oxidation under physiological conditions. Thus, most non-enzymatic glucose biosensors reported in the literature use alkaline media to detect glucose, while the physiological pH of blood is 7.4. This is particularly relevant for the electrodes made of Cu, Ni, metal oxides, and carbon nanomaterials, despite their excellent sensitivity. Thus, these electrodes cannot be used to directly diagnose diabetes without a preliminary blood preparation. Another major challenge in non-enzymatic glucose biosensors is the selectivity of these devices. The GOx enzyme is specific for glucose detection in enzymatic glucose biosensors; however, in non-enzymatic glucose biosensors, interfering species in the blood, such as dopamine, uric acid, and ascorbic acid, may react with the electrode surface and cause errors in blood glucose detection. Consequently, it is important to choose a catalyst that has excellent electrocatalytic properties. There is ongoing research on non-enzymatic biosensors, but further research on electrodes with different electro-catalysts is needed, for their commercialization as the fourth generation of electrochemical glucose biosensors. Fabrication cost is the major concern in the commercialization of non-enzymatic electrodes. As a result, relatively less research has been done on this type of CNF-based biosensor. It would be beneficial to conduct further studies using new nanostructured materials as catalysts for reactions. However, in the last decade, this type of biosensor has attracted the attention of many researchers. Non-enzymatic glucose biosensors are still in the R&D stage and have not yet reached the commercialization stage. The most common type of glucometer available on the market is the enzymatic biosensor. In this regard, it is hoped that researchers in this field will develop fourth-generation commercial glucometers based on non-enzymatic biosensors.

## Figures and Tables

**Table 1:** CNF-based non-enzymatic glucose biosensors and their detection performances.

Electrode	Sensitivity (μA·mM^−1^·cm^−2^)	LR	LOD	Applied Potential (V)	Ref.
		(mM)	(mM)		
Ni(OH)_2_/ECNF	1342.2	5 × 10^−3^–13.05	10^−4^	0.55	[106]
APBA ^a^ @CoFe_2_O_4_/CNFs	318	10^−2^–3.52	3.25 × 10^−4^	0.55	[107]
Ni_2_CoS_4_/CNF/GCE ^b^	–	5 × 10^−6^–70 × 10^−6^	25 × 10^−8^	0.54	[108]
Ni_0.66_Co_0.33_(OH)_2_/CNF/GCE	1470	10^−3^–2	3 × 10^−5^	0.5	[109]
Co_3_O_4_/CNF	–	10^−6^–10^−2^	10^−6^	0.55	[110]
Ni/CNF	6393.4	1.25 × 10^−4^–1.273 × 10^−2^	5 × 10^−5^	0.359	[111]
Co/CNFs/GCE	97	0.5–3.5	5 × 10^−2^	0.6	[112]
FeCo/CNFs/GCE	196	0.2–10	10^−2^		[112]
NiCo/CNFs/GCE	141	0.1–10	2 × 10^−2^		[112]
MnCo/CNFs/GCE	36	0.5–7	5 × 10^−2^		[112]
CuCo/CNFs/GCE	507	0.02–11	10^−3^		[112]
NiMoO_4_/CNF	301.77	3 × 10^−4^–4.5	5 × 10^−5^	0.55	[113]
NiP_2_/CNF	–	4 × 10^−4^–6.4 × 10^−3^ and 0.5–1.5	13 × 10^−5^	0.5	[114]
Pt/CNF_39nm_	2.03	0.3–17	3.3 × 10^−2^		[114]
Pt/CNF_158nm_	1.63	0.3–15	5.28 × 10^−2^	0.05	[115]
Pt/CNF_309nm_	1.01	0.3–13	7.03 × 10^−2^		[115]
MnO_2_–Co_3_O_4_/ECNF	1159	5 × 10^−3^–10.9	3 × 10^−4^	0.55	[116]
CuONPs–CNFs	2739	5 × 10^−4^–11.1	2 × 10^−4^	0.5	[117]
Ni(OH)_2_/CNFs/GCE	1038.64	10^−3^–1.2	76 × 10^−5^	0.45	[118]
NiCo_2_S_4_/ECNF	7431.96	5 × 10^−4^–3.571	167 × 10^−6^	0.5	[119]
Ni–CoO/CNF/GCE	–	25 × 10^−5^–0.6	3 × 10^−5^	0.5	[120]
Ni/CNFs	–	2 × 10^−3^–5	57 × 10^−5^	0.55	[121]
Ni/CNF paste	420.4	2 × 10^−3^–2.5	10^−3^	0.6	[122]
Pd−Ni/ECNF	–	3 × 10^−5^–0.8	7 × 10^−6^	0.4	[123]
NiCo_2_O_4_/ECNF	1947.2	5 × 10^−3^–19.175	1.5 × 10^−3^	0.55	[124]
CuO/N–CNFs ^c^	968 and 484	0.25–2 and 2–4	–	0.5	[125]
CuO/NiO/ACNF ^d^	247	2.5 × 10^−4^–5	1.46 × 10^−4^	0.55	[126]
0.5 Ni/ECNF–5 h	610.6	2–10	0.73	0.5	[61]
NiO/ECNF	557.68	2–10	0.85	0.5	[61]
0.5 Co/ECNF–5 h	236.85	2–10	0.61	0.6	[61]
Co_3_O_4_/ECNF	475.72	2–10	0.82	0.6	[61]
Ni_70_Co_30_/ECNF–5 h	498.53	2–10	0.91	0.55	[61]
NiCo_2_O_4_/ECNF	536.5	2–10	0.93	0.55	[61]
CuSn/CNFs	291.4	10^−4^–9	8 × 10^−5^	0.7	[127]
Ni/CNFs1400 ^e^	7404	2.1 × 10^−2^–0.6	4.97 × 10^−5^	0.55	[128]
Ni–MOF ^f^/CNF	9457.5	10^−2^–3	5.3 × 10^−5^	0.7	[129]
Pt/CNF	0.52	2–20	–	0.55	[130]
CuO/rGO ^g^/CNF/GCE	912.7	10^−3^–5.3	10^−4^	0.6	[131]
PTBA ^h^/CuCo_2_O_4_/CNFs/GCE	2932 and 708	10^−2^–0.5 and 0.5–1.5	15 × 10^−5^	–	[132]

a: Aminophenylboric acid; b: glassy carbon electrode; c: nitrogen-doped carbon nanofibers; d: activated carbon nanofiber; e: CNF with a carbonization at temperature of 1400 °C; f: Metal–organic framework; g: reduced graphene oxide; h: poly(thiophene-3-boronic acid).

## Data Availability

Not applicable.
